# Fabrication of a Flexible Micro CO Sensor for Micro Reformer Applications

**DOI:** 10.3390/s101210701

**Published:** 2010-11-30

**Authors:** Chi-Yuan Lee, Chi-Chung Chang, Yi-Man Lo

**Affiliations:** Department of Mechanical Engineering, Yuan Ze Fuel Cell Center, Yuan Ze University, Taoyuan, Taiwan; E-Mails: s975055@mail.yzu.edu.tw (C.-C.C.); s995048@mail.yzu.edu.tw (Y.-M.L.)

**Keywords:** MEMS, flexible micro CO sensor, micro reformer

## Abstract

Integration of a reformer and a proton exchange membrane fuel cell (PEMFC) is problematic due to the presence in the gas from the reforming process of a slight amount of carbon monoxide. Carbon monoxide poisons the catalyst of the proton exchange membrane fuel cell subsequently degrading the fuel cell performance, and necessitating the sublimation of the reaction gas before supplying to fuel cells. Based on the use of micro-electro-mechanical systems (MEMS) technology to manufacture flexible micro CO sensors, this study elucidates the relation between a micro CO sensor and different SnO_2_ thin film thicknesses. Experimental results indicate that the sensitivity increases at temperatures ranging from 100–300 °C. Additionally, the best sensitivity is obtained at a specific temperature. For instance, the best sensitivity of SnO_2_ thin film thickness of 100 nm at 300 °C is 59.3%. Moreover, a flexible micro CO sensor is embedded into a micro reformer to determine the CO concentration in each part of a micro reformer in the future, demonstrating the inner reaction of a micro reformer in depth and immediate detection.

## 1. Introduction

Integration of a reformer and a proton exchange membrane fuel cell (PEMFC) is problematic due to the gas from the reforming process which contains a slight amount of carbon monoxide. Carbon monoxide poisons the catalyst of the proton exchange membrane fuel cell subsequently degrading the fuel cell performance, and necessitating the sublimation of the reaction gas before supplying it to fuel cells. Additionally, detecting extremely low concentrations of CO in pure H_2_ or H_2_-rich gases in a fuel cell is important. Given the well known poisoning of platinum by CO, maintaining satisfactory performance in a PEMFC requires avoiding CO in the fuel gas and devising CO sensors that will operate under extreme conditions.

Fukuie [1] found that incorporating La_2_O_3_ and Au can successfully cause the activation of CO inherent in gold and make it possible to detect CO selectively and stably. Williams [2] fabricated a CO sensor using tin oxide (SnO_2_), in which platinum has a lower reaction temperature, faster response time and shorter recovery time.

Horrillo [3] investigated not only how catalysts (Pt and Pd) affect tin-oxide films, but also how Pt and Pd catalysts affect films prepared by reactive sputtering and screen-printing based on an analysis of their respective roles. The catalyst particles compensate for the holes between the grains of the tin-oxide films prepared by screen-printing, subsequently increasing the stability and reproducibility of the films and achieving an excellent CO detection capability at low concentrations. According to their results, Pt and Pd catalysts increased the sensitivity and diminish the times of recuperation.

Habibzadeh [4] studied how Sm_2_O_3_ influences the sensitivity and selectivity of SnO_2_-based sensors for the selective detection of CO. Sm_2_O_3_ acts as a crystal growth inhibitor and decreases SnO_2_ sizes. Additionally, oxygen adsorption was increased by doping Sm_2_O_3_, subsequently increasing the depletion of layer thickness.

Izu [5] fabricated CO sensor elements by connecting two cerium oxide (ceria) thick films. The cerium oxide thick film sintered at 950 °C displayed a better response for CO gas and lower resistance. Moreover, the connected sensor element with Pt/alumina catalyst fabricated in that study exhibited better sensitivity.

Pijolat [6] studied a samarium doped ceria (SDC) sensor, *i.e.*, a potentiometric sensor working with an electrode dissymmetry Au/Pt. This sensor detects low CO concentrations in H_2_-rich atmosphere for PEMFC applications.

Despite various CO sensor applications [7–9], micro reformer-related applications have received less attention. The micro CO sensor based on MEMS technology is characterized by its small volume, light mass and high precision. In this study, a flexible micro CO sensor is embedded into a micro reformer to determine the CO concentration in each part of a micro reformer in the future, conferring the inner reaction of a micro reformer depth and immediate detection capability.

## 2. Methodology

By adopting a semiconductor sensor, the CO sensor in this study uses reductive gas and oxygen adsorption at the surface of a gas-sensitive thin film, due to the relative ease that an oxygen atom has in seizing electrons inside the material and becoming an oxygen ion after adsorbing on the surface of gas-sensitive thin film, ultimately decreasing the number of electrons inside the gas-sensitive thin film and increasing its resistance. When the surface of a gas-sensitive thin film contacts with a reductive gas, e.g., carbon monoxide, the reductive gas reacts with the oxygen ion that adsorbs on the surface of gas-sensitive thin film, ultimately releasing electrons to a gas-sensitive thin film and decreasing the resistance of a material as indicated by the following scheme [10]:

(1) $$\text{CO}+1/2\;{\text{O}}_{2}\to {\text{CO}}_{2}$$

The gas-sensitive thin film in this study uses SnO_2_, which is characterized by its high sensitivity to hydrocarbons, stable chemical properties and high fabrication compatibility, all of which account for its high suitability in a gas-sensitive thin film. The extensive use of oxide in the SnO_2_ base allows SnO_2_ to work at a low operational temperature, as well as have a high sensitivity [10]. Gas-sensitive sensors are characterized by their sensitivity, stability, repeatability and selectivity. At an appropriate operational temperature, adsorption and desorption of oxygen occur, in which the difference in temperature leads to a difference of sensitivity. Additionally, the reaction rate increases when selecting an appropriate operational temperature, subsequently decreasing the response time between SnO_2_ thin film and reductive gas. The measurement results are influenced by the adsorption and desorption at the material surface. Notably, appropriately adding a catalyst can decrease the reaction temperature, as well as raise the response time, recovery time and selectivity. Obviously, a high sensitivity, quick response time and reuse are all essential features for a gas-sensitive sensor.

## 3. Fabrication of Flexible Micro CO Sensors

**Figure 1.**
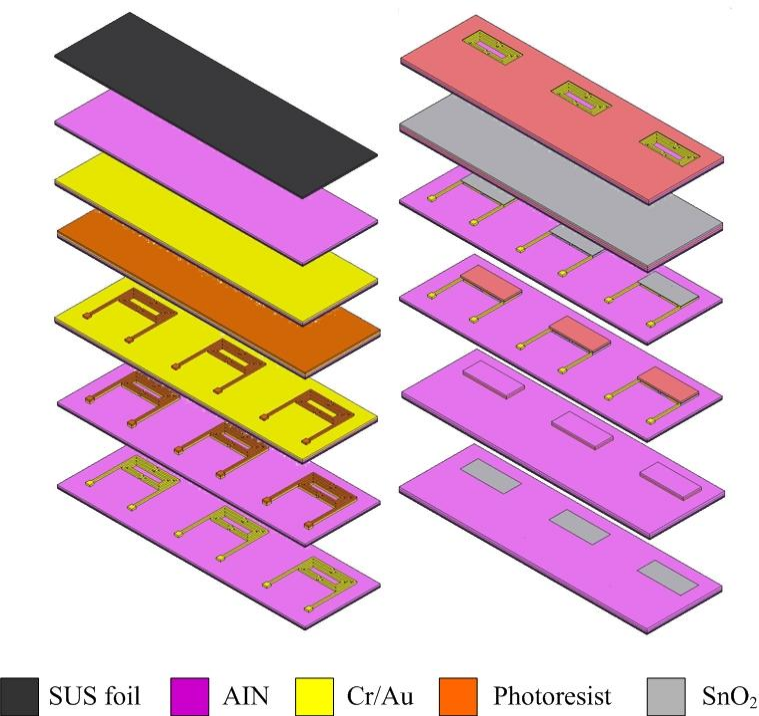
Fabrication flowchart of a flexible micro CO sensor.

Figure 1 shows the fabrication of the flexible micro CO sensors. The first step involves rinsing stainless steel foil with Piranha etchant and controlling the temperature to remove the oxide on the surface.

**Figure 2.**
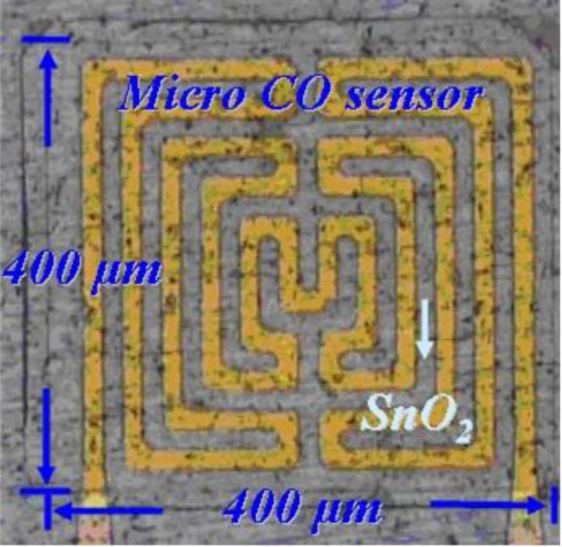
Optical microscopy image of a micro CO sensor.

After cleaning, the stainless steel foil is soaked in acetone and placed in an ultrasonic cleaner, followed by soaking in methanol and washing with DI-water. The second step involves using RF sputter to sputter 10,000 Å of AlN on the stainless steel foil to form the isolation layer. The third step involves using the E-beam evaporator to evaporate 400 Å of chromium and 2,000 Å of gold on the aluminum nitride to be a sticking layer and electrode layer, respectively. The fourth step involves using a spin coater to coat HMDS to be the sticking layer for a photoresist, followed by coating of AZ-4620 photoresist. The fifth step involves using an aligner to transit the pattern of a photoresist, in which the developer is used to devise the required shape. The sixth step involves using etchant (Type-TFA) to etch the gold and another etchant (Cr-7T) to etch the chromium. The seventh step involves removing the photoresist. The eighth step involves coating the photoresist on stainless steel foil to be a sputtering mask, followed by transiting the pattern of photoresist and developing the required shape. The ninth step involves using RF sputter to sputter SnO_2_ on the stainless steel foil to become the gas sensitive layer. The tenth step involves lifting off the photoresist with stripper (Remove 1165) at 80 °C. The eleventh step involves coating a photoresist on the stainless steel foil to be a sputtering mask, transiting the pattern of photoresist and developing the required shape. The twelfth step involves using RF sputter to sputter aluminum nitride on the stainless steel to become an isolation layer. Following soaking of the stainless steel foil in an etchant (Remove 1165) to lift off and etch aluminum nitride, the micro CO sensor is finished. Figure 2 schematically depicts a CO sensor.

## 4. Results and Discussion

### 4.1. Measurement System of a Micro CO Sensor

**Figure 3.**
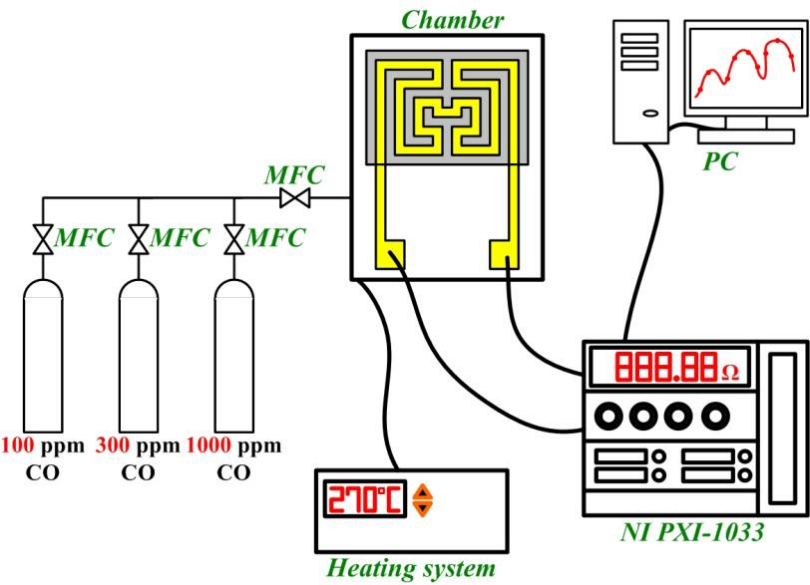
Calibration system of CO sensor.

Adjusting the micro CO sensor initially involves heating the sensitive element to a working temperature, followed by infusion with air until the resistance achieves a constant value, subsequently infusing 100, 300, 1,000 ppm of CO/N_2_ mixing gas into the micro reformer. A mass flow controller (MFC) is used to control the flow and a NI PXI-1033 as well as a computer are used to record the resistance. Response time is determined by the curve between time and resistance by infusing air and CO/N_2_ mixing gas. The CO sensor calibration system is shown in Figure 3.

### 4.2. Effect of SnO_2_ Thickness on Micro CO Sensor at Various Temperatures

**Figure 4.**
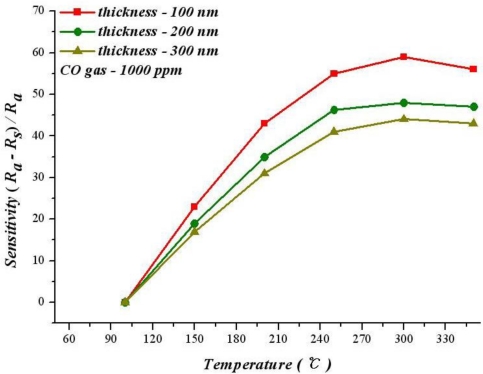
Relation between temperature and sensitivity for various SnO_2_ thin film thicknesses.

Figure 4 shows the relationship between operating temperature and sensitivity with different SnO_2_ thicknesses at 1,000 ppm of CO. According to this figure, the sensitivity increases with temperature at 100–300 °C.

**Figure 5.**
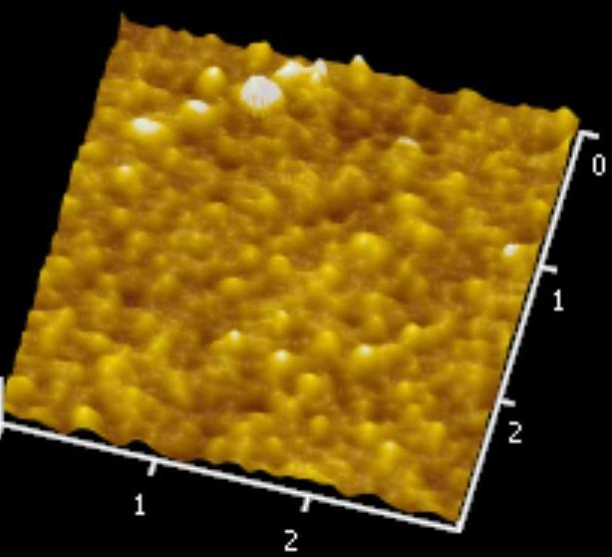
AFM photo of 100 nm SnO_2_ thin film.

**Figure 6.**
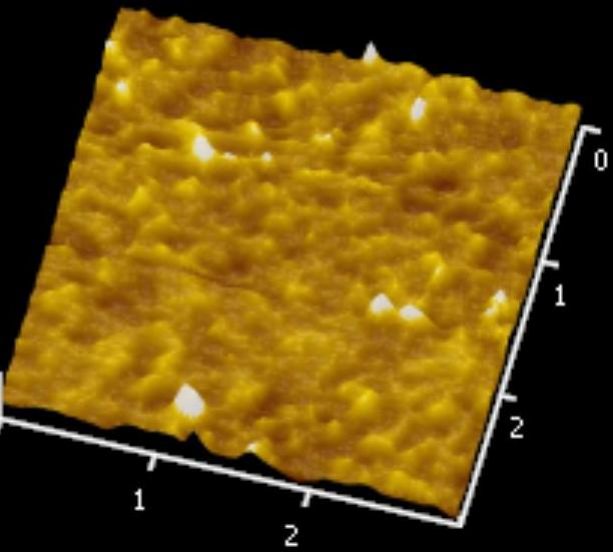
AFM photo of 300 nm SnO_2_ thin film.

**Figure 7.**
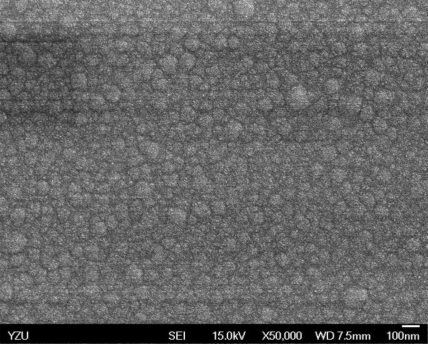
SEM photo of 100 nm SnO_2_ thin film.

**Figure 8.**
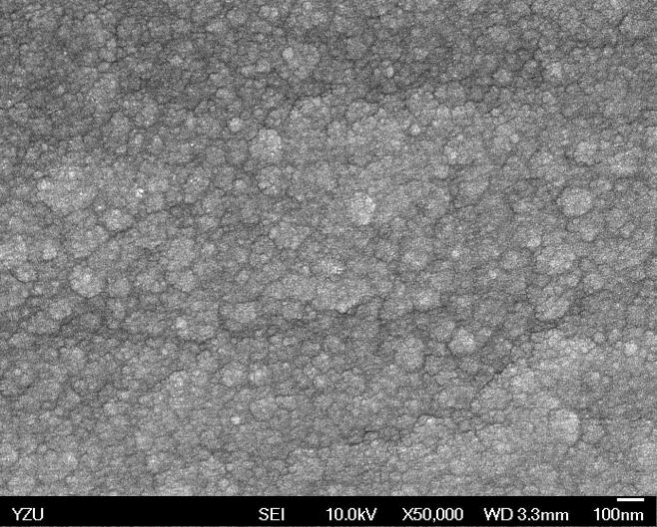
SEM photo of 200 nm SnO_2_ thin film.

**Figure 9.**
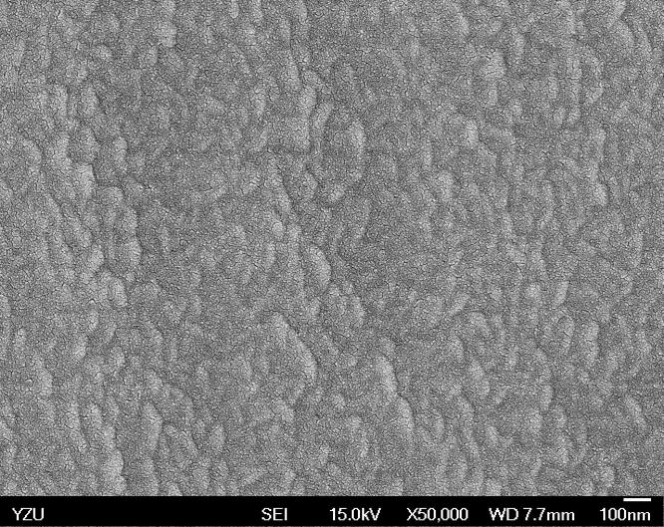
SEM photo of 300 nm SnO_2_ thin film.

Figures 5 and 6 illustrate the atomic force microscope (AFM) photographs of the SnO_2_ thin film, while Figures 7 to 9 display scanning electron microscope (SEM) photographs of the SnO_2_ thin film. According to these figures, 100 nm of SnO_2_ thin film has a smaller grain, producing a larger surface, allowing it to adsorb more oxygen on the surface, and achieving a higher sensitivity.

As an exothermic reaction, the adsorption reaction is unfavorable for the reaction after the temperature exceeds a critical temperature after infusing CO. Therefore, the optimum sensitivity can be obtained at a specific temperature. Figure 4 reveals that the best sensitivity of 100 nm SnO_2_ thin film operating at 300 °C is 59.3%. However, when the thicknesses are 200 nm and 300 nm, operating at 350 °C leads to best sensitivities of 48.3% and 43.5%.

### 4.3. Effect of SnO_2_ Thickness on a Micro CO Sensor at 280 °C

**Figure 10.**
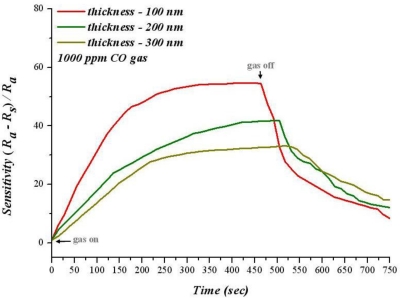
Relation between temperature and sensitivity for various SnO_2_ thin film thicknesses (T = 280 °C).

When the temperature is lower than 300 °C, sensitivity decreases with increasing film thickness at the same temperature. For sensitivity analysis, the adsorption of gas sensitive thin film is used to change the resistance to gas; a larger reaction area causes a greater adsorbption of oxygen ions; in addition, the resistance increases when infusing a reductive gas. Consequently, increasing the reaction area can raise the sensitivity. Figure 10 shows the diagram of sensitivity and time variation for 1,000 ppm of CO at 280 °C. When carbon monoxide is infused, sensitivity increases as time proceeds. Moreover, the rate of increasing of 100 nm SnO_2_ thin film sensitivity is faster than that of 200 nm and 300 nm films; the response time is faster as well.

When carbon monoxide stops infusing, the recovery time of 100 nm SnO_2_ thin film is faster than for 200 nm and 300 nm film. Hence, when carbon monoxide disappears, carbon dioxide has a different desorption ratio owing to the different thicknesses of SnO_2_ thin film. Moreover, 100 nm of SnO_2_ thin film has a higher recovery time because CO_2_ more easily desorbed, subsequently adsorbing more oxygen ions on the surface.

### 4.4. Analysis of Reproducibility and Stability

**Figure 11.**
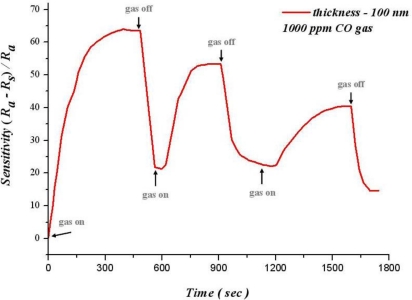
Analysis of reproducibility and stability (T = 300 °C).

Reproducibility refers to the unity of output signals after successive testing. A normal gas sensor not only has a high sensitivity, but also reproducibility and stability. Figure 11 shows the relation between operating time and sensitivity for 100 nm of SnO_2_ thin film at 300 °C in 1,000 ppm of carbon monoxide. This figure reveals that when carbon monoxide is removed, sensitivity decreases quickly. Although carbon monoxide is infused in 600 seconds, the sensitivity is not restored to the initial value. Furthermore, sensitivity decreases with increasing cycle number (when it cycles three times, sensitivity decreases from 65.3% to 39.2%).

**Figure 12.**
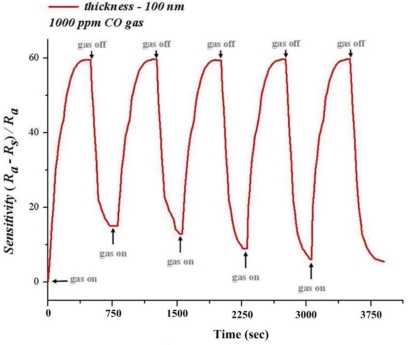
Stability testing in 1,000 ppm of CO (T = 270 °C).

Sensitivity cannot remain in a steady state at 300 °C. However, when the temperature is reduced to 270 °C, the sensitivity has a better reproducibility and stability, as shown in Figure 12. The sensitivity of 100 nm thin film can be maintained at 59.5% at the 750th second for 1,000 ppm of CO. Thus, the sensitivity has a higher better reproducibility and stability at 270 °C.

**Figure 13.**
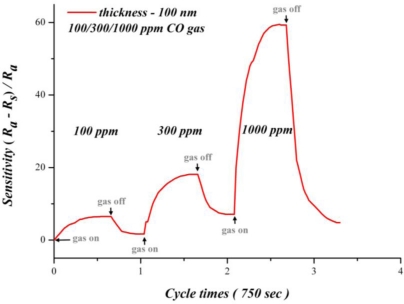
Testing for various CO concentrations (270 °C).

Figure 13 shows how different CO concentrations affect the sensitivity of 100 nm SnO_2_ film. According to this figure, the sensitivity increases with the CO concentration. An increasing difference of CO concentration also increases the variation of sensitivity. This finding suggests that the SnO_2_ thin film has a good sensitivity for different CO concentrations. At different concentrations of carbon monoxide, removing carbon monoxide significantly decreases sensitivity, which remains uninfluenced by the environment. This figure also reveals that sensitivity easily becomes saturated with a decreasing concentration of carbon monoxide. However, the reaction time is not obviously shortened; it is the same due to the relation between reaction time and operating time. Although SnO_2_ thin film has a faster response time and best sensitivity at 300 °C, the reproducibility and chronic stability are not as good. For reproducibility and chronic stability, 270 °C is a preferred operating temperature.

### 4.5. Effect of Heat Treatment on Thin Film Sensitive Characteristics

**Figure 14.**
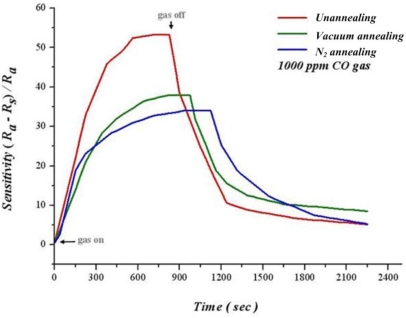
Analysis of different heat treatments to CO sensitivity.

Figure 14 shows 100 nm of as-deposited film processing heat treatment in oxygen and vacuum separately at 400 °C for 1 hour, as well as dynamic analysis of sensitivity at 250 °C, 1,000 ppm of CO. The thin film has not been heat treated with the highest sensitivity of 54.11% after infusing CO for 350 seconds. Additionally, the thin film after heat treatment is thinner than before heat treatment. We can infer that the thin film after heat treatment becomes more flat, leading to a decreased adsorption of oxygen. Consequently, the sensitivity of thin film after heat treatment is lower than that before heat treatment. Although lower after heat treatment, sensitivity of thin films increases steadily before saturation.

### 4.6. Effect of Catalyst on Response Time

**Figure 15.**
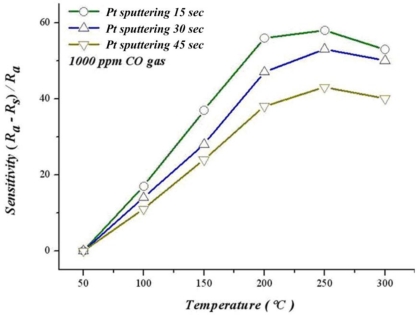
Relation between temperature and sensitivity during various platinum deposition times.

Figure 15 shows different sputtering times for platinum sludging on SnO_2_ thin film, the relation between sensitivity and operating temperature in 1,000 ppm of CO. When sputtering Pt for 15 seconds, 30 seconds and 45 seconds, our results indicate that when the temperature approaches 250 °C, sensitivity decreases with a rising temperature. This temperature may be related to a lower catalyst activity. When the temperature increases to 300 °C, sensitivity decreases due to the difficulty to proceed with the reaction when the temperature exceeds a critical temperature. The optimum sensitivity for adding a catalyst ranges from 200 °C to 250 °C. Moreover, sensitivity of sputtering 45 seconds does not increase when adding more catalyst because platinum quarantines the CO and SnO_2_ thin films.

**Figure 16.**
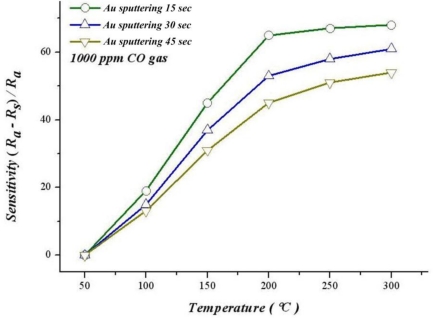
Relation between temperature and sensitivity during different gold deposition intervals.

Moreover, according to Figure 16, the best sensitivity at 300 °C of sputtering Au for 15 seconds, 30 seconds and 45 seconds are 68.3%, 61.3% and 54.2%. The sensitivity of sputtering Au for 15 seconds at 150 °C is 45.74%, *i.e.*, significantly higher than the thin film that does not have gold added (13.33%). This finding suggests that adding gold can significantly increase sensitivity and response time.

According to previous studies [11–14], the oxidation activity of gold is significantly higher than that of platinum, ruthenium and palladium; in addition, the catalyst life is also longer. This finding explains why sputtering Au gives a better response time and sensitivity than sputtering Pt, and sensitivity of gold increasing with a rising temperature.

## 5. Conclusions

This study describes the successful fabrication of a micro CO sensor with MEMS technology, in which gas sensitivity influences CO. Additionally, the micro CO sensor could be embedded into a micro reformer to evaluate the CO concentration in each part of the micro reformer in the future, demonstrating the inner reactions of the micro reformer in depth with immediate detection capability.
